# Long-Term Outcomes of Endoscopic Ultrasound-Guided Gallbladder Drainage for Acute Cholecystitis in Non-Surgical Candidates: A Multicenter Retrospective Study

**DOI:** 10.3390/jcm15103621

**Published:** 2026-05-08

**Authors:** Kei Harada, Kazuya Miyamoto, Kazuyuki Matsumoto, Kosaku Morimoto, Eijiro Ueta, Yutaka Akimoto, Nao Hattori, Taisuke Obata, Ryosuke Sato, Akihiro Matsumi, Hiroyuki Terasawa, Yuki Fujii, Daisuke Uchida, Shigeru Horiguchi, Koichiro Tsutsumi, Motoyuki Otsuka

**Affiliations:** 1 Department of Gastroenterology and Hepatology, Okayama University Hospital, 2-5-1 Shikata-cho, Okayama 700-8558, Japan; keeeeeei66@gmail.com (K.H.); nhattori1007@gmail.com (N.H.); tobata20023@gmail.com (T.O.); rsato0731@gmail.com (R.S.); me17053@s.okadai.jp (H.T.); d.uchida0309@gmail.com (D.U.); horiguchis@gmail.com (S.H.); tsutsumi@cc.okayama-u.ac.jp (K.T.); otsukamoto@okayama-u.ac.jp (M.O.); 2 Department of Endoscopy, Okayama University Hospital, 2-5-1 Shikata-cho, Okayama 700-8558, Japan; matsumoto.k@okayama-u.ac.jp (K.M.); akihiro.matsumi.gastro@gmail.com (A.M.); y_f1105@yahoo.co.jp (Y.F.); 3 Department of Internal Medicine, Tsuyama Chuo Hospital, 1756 Kawasaki, Tsuyama 708-0841, Japan; pabaimar2110@yahoo.co.jp; 4 Department of Gastroenterology, National Organization Iwakuni Clinical Center, 1-1-1 Atago-machi, Iwakuni 740-8510, Japan; eu.europe2012@gmail.com; 5 Department of Gastroenterology, Japanese Red Cross Okayama Hospital, 2-1-1 Aoe, Okayama 700-8607, Japan; qttsp974@gmail.com

**Keywords:** cholecystitis, drainage, endosonography, gallbladder

## Abstract

**Background/Objectives:** Endoscopic ultrasound-guided gallbladder drainage (EUS-GBD) is a minimally invasive alternative for managing acute cholecystitis in patients who are unsuitable for surgery. Although its short-term efficacy is well-established, its long-term outcomes, especially in patients with malignancy-associated cholecystitis, remain unclear. **Methods:** This multicenter, retrospective study included 139 patients who underwent EUS-GBD with a plastic stent for inoperable acute cholecystitis between January 2010 and October 2023. Patients were divided into two groups: a malignant group (*n* = 60) with cystic duct obstruction caused by cancer invasion or self-expandable metal stents, and a benign group (*n* = 79) with calculous or acalculous cholecystitis. The outcomes assessed included cholecystitis recurrence, time to recurrence, adverse events, and risk factors for recurrence. **Results:** Technical success was achieved in all patients, with an overall clinical success rate of 94.6%. Cholecystitis recurred significantly more frequently in the malignant group than in the benign group (13.3% vs. 2.5%; *p* = 0.015). Univariate analysis identified malignancy as a significant risk factor of recurrence (odds ratio, 5.92; *p* = 0.028). **Conclusions:** EUS-GBD is a safe and effective long-term treatment for cholecystitis in non-surgical candidates. However, malignancy-associated cholecystitis carries a high risk of recurrence, warranting careful follow-up and individualized management.

## 1. Introduction

Acute cholecystitis is a common gastrointestinal disease that can be fatal if the treatment is delayed. Surgical cholecystectomy is the first-line treatment [1,2]. However, advanced age and significant comorbidities may limit surgical candidacy, as these factors are associated with an increased risk of perioperative morbidity and mortality related to surgery and anesthesia [3]. In addition, acute cholecystitis caused by malignant tumor invasion of the cystic duct, including unresectable biliary tract or pancreatic cancer, requires a nonsurgical approach.

Endoscopic ultrasound-guided gallbladder drainage (EUS-GBD) has emerged as a minimally invasive and effective alternative for patients who are unsuitable for surgery. Compared to percutaneous transhepatic gallbladder drainage (PTGBD), EUS-GBD offers advantages such as greater patient comfort and reduced hospital readmissions [4,5,6,7]. It also demonstrates higher technical success rates and fewer AEs such as cholangitis or post-ERCP pancreatitis than endoscopic transpapillary gallbladder drainage (ETGBD) [8,9,10]. Furthermore, EUS-GBD is a valuable option for managing acute cholecystitis caused by self-expandable metal stents (SEMSs) placed in malignant biliary strictures [11,12,13,14].

Although the short-term efficacy and safety of EUS-GBD have been well-reported [15], data on its long-term outcomes remain limited. In particular, no study has evaluated the long-term results of EUS-GBD in patients with acute cholecystitis due to cystic duct obstruction caused by malignant tumor invasion or SEMSs. As this subgroup is increasingly encountered in clinical practice, clarification of the long-term effectiveness of EUS-GBD in such cases is critical to guide optimal management.

Therefore, this multicenter retrospective study aimed to evaluate the long-term outcomes of EUS-GBD in nonsurgical candidates with acute cholecystitis, with a particular focus on recurrence rates. By comparing patients with malignant and benign cholecystitis, we sought to identify the risk factors for recurrence and clarify the clinical utility of EUS-GBD in each context. These findings may contribute to individualized treatment strategies.

## 2. Methods

### 2.1. Patients

This retrospective study investigated patients who underwent EUS-GBD at four tertiary care referral centers between 1 January 2010, and 31 October 2023. The inclusion criteria were as follows: (1) age ≥ 18 years and (2) poor surgical candidates. The exclusion criteria were as follows: (1) cardiopulmonary failure precluding endoscopic procedures, (2) difficulty in discontinuing antithrombotic agents or presence of significant coagulation disorders, (3) surgically modified gastrointestinal anatomy, (4) substantial ascites, and (5) unavailability of detailed clinical or procedural data. This retrospective study was performed in accordance with the Declaration of Helsinki and approved by the Okayama University Graduate School of Medicine, Dentistry and Pharmaceutical Sciences, Okayama University Hospital, and the Ethics Committee Review Board for Human Research (approval no. 2404-013; approved on 12 April 2024).

### 2.2. EUS-GBD Procedure

A linear-array echoendoscope (Olympus Medical Systems, Tokyo, Japan) was inserted and the gallbladder was punctured from the duodenum or stomach using a 19-gauge needle. After placing a 0.025-inch guidewire in the gallbladder, the tract was dilated using a bougie dilator, balloon catheter, or electrocautery dilator. A 7Fr double-pigtail plastic stent (PS) was placed across the tract. In patients with concomitant choledocholithiasis, transpapillary stone removal was performed before EUS-GBD.

### 2.3. Follow-Up

Patients were advised to seek medical attention promptly if symptoms such as abdominal pain occurred. If adverse events (AEs), including recurrent cholecystitis, were suspected, diagnostic tests, such as blood tests, ultrasound, CT, or MRCP, were performed. Information was collected from the medical records of the patients who regularly visited the hospital. Those who had not visited for over 6 months were followed up via telephone.

### 2.4. Outcomes and Definitions

The primary endpoint was cholecystitis recurrence rate, defined as the appearance of clinical symptoms after initial clinical success. The secondary endpoints included time to recurrence, technical and clinical success rates, procedure time, and risk factors for recurrence. The time to recurrence was determined based on the time of PS placement. Technical success was defined as successful stent placement in the gallbladder, and clinical success was defined as the resolution of symptoms and laboratory abnormalities without the need for additional drainage procedures. Cholecystitis and cholangitis were diagnosed and graded according to the Tokyo Guidelines 2018 [16,17]. AEs were classified and graded according to the American Society for Gastrointestinal Endoscopy (ASGE) criteria [18]. Early AEs occurred within 14 days after the procedure, and late AEs occurred after 14 days.

Patients were divided into two groups. The malignant group included patients with acute cholecystitis due to cystic duct obstruction caused by cancer invasion or SEMS placement. Cystic duct obstruction was evaluated using contrast-enhanced computed tomography. All SEMSs placed were covered metal stents. The benign group included patients with other types of acute cholecystitis, such as calculous or acalculous cholecystitis, unrelated to cancer invasion or SEMSs, and no patients in the benign group underwent SEMS placement.

### 2.5. Statistical Analysis

Continuous variables are presented as medians and interquartile ranges (IQRs) and were compared using the Mann–Whitney U test. Categorical variables were compared using the chi-squared test. Univariate logistic regression analysis was performed to identify significant risk factors for recurrence. Statistical significance was defined as *p* < 0.05. The cumulative incidence of recurrent cholecystitis was estimated using the Kaplan–Meier method and compared between groups using the log-rank test. All analyses were conducted using the JMP Pro 17 software (SAS Institute Inc., Cary, NC, USA).

## 3. Results

### 3.1. Patient Characteristics

**Figure 1:**
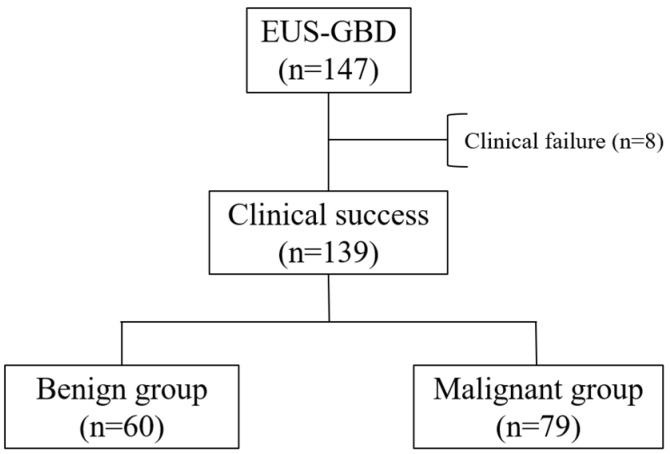
Study flow diagram; EUS-GBD: Endoscopic ultrasound-guided gallbladder drainage.

Of the 147 patients, 8 patients who did not achieve clinical success (5 in the malignant group and 3 in the benign group) were excluded. Among these, 6 patients improved following additional placement of PS, whereas 2 patients died due to worsening cholecystitis. In total, 139 patients were included in this study. (Figure 1). The characteristics of the 139 patients with 60 in the malignant group and 79 in the benign group are summarized in Table 1. Compared to the benign group, patients in the malignant group had a significantly lower median age (78 vs. 87 years, *p* < 0.0001). The malignant group also had fewer patients with an ASA-PS > 3 (32% vs. 66%, *p* < 0.0001) and less frequent use of antithrombotic agents (10% vs. 43%, *p* = 0.0007). Gallbladder stones were significantly more common in the benign group (82% vs. 18%, *p* < 0.0001) and severe cholecystitis occurred only in the benign group (0% vs. 11%, *p* = 0.0069). The median follow-up duration was significantly shorter in the malignant group (116 vs. 307 days, *p* = 0.0002), likely reflecting the prognosis of the underlying disease.

### 3.2. Outcomes of EUS-GBD

**Figure 2:**
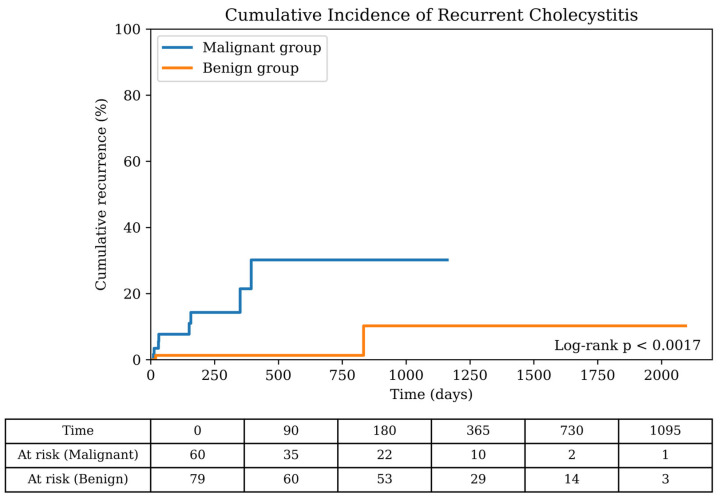
Cumulative incidence of recurrent cholecystitis after EUS-GBD. The cumulative incidence of recurrent cholecystitis after EUS-GBD in the benign and malignant groups was estimated using the Kaplan–Meier method. The numbers at risk are shown below the x-axis. EUS-GBD: Endoscopic ultrasound-guided gallbladder drainage.

The procedural outcomes of EUS-GBD are shown in Table 2. The technical success rate was 100% and the clinical success rate was 94.6% (139/147). The cholecystitis recurrence rate was significantly higher in the malignant group (13.3% [8/60] vs. 2.5% [2/79], *p* = 0.015). Among the eight recurrent cases in the malignant group, six were attributed to cancer invasion, whereas two were associated with SEMSs. Kaplan–Meier analysis showed that the cumulative incidence of recurrent cholecystitis was significantly higher in the malignant group than in the benign group (*p* = 0.0017) (Figure 2). The median time to recurrence was 91 days for the malignant group and 426 days for the benign group. Among patients with recurrent cholecystitis, the initial severity was mild in four cases and moderate in six cases. Reintervention was performed in nine of ten cases, including additional stent placement in four, stent exchange in four, and PTGBD in one. One patient with calculous cholecystitis was conservatively treated. All the patients showed clinical improvement (Table S1). No patients underwent elective cholecystectomy or stent removal during the follow-up period in either the malignant or benign groups.

### 3.3. Risk Factors for Recurrent Cholecystitis

The detailed results of the univariate analysis are presented in Table 3. Univariate analysis revealed that malignancy was significantly associated with recurrence (odds ratio, 5.92; 95% CI: 1.21–29.01; *p* = 0.028). Other variables—advanced age (>84 years), male sex, use of antithrombotic agents, ASA-PS > 3, and presence of gallstones—were not significantly associated with recurrence. Notably, none of the patients with severe cholecystitis experienced a recurrence. Although univariate analysis yielded meaningful findings, the small number of recurrent cases (*n* = 10) precluded multivariate analysis, and the results should be interpreted with caution.

### 3.4. AEs

Early AEs occurred in 10.7% of the patients, with no significant difference between the two groups (*p* = 0.214). Biliary peritonitis occurred in 11 patients (7.9%), with 7 cases being mild, 3 moderate, and 1 severe, all of which were managed with conservative treatment. Stent migration occurred in four patients (2.8%), all of whom were successfully treated endoscopically.

Late AEs, excluding recurrent cholecystitis, were observed in two patients (1.4%), both presenting with mild cholangitis that resolved with endoscopically treatment. These events occurred only in the benign group, although the difference was not statistically significant (*p* = 0.400). This was presumably caused by the migration of gallstones into the common bile duct.

## 4. Discussion

This study provides important insights into long-term outcomes of EUS-GBD in patients with acute cholecystitis. A comparison of outcomes between patients with acute cholecystitis due to cystic duct obstruction caused by cancer invasion or SEMS placement (malignant group) and those with calculous or acalculous cholecystitis (benign group) revealed that the recurrence rate of cholecystitis was significantly higher in the malignant group (13.3% vs. 2.5%, *p* = 0.015).

Cholecystectomy remains the standard treatment for acute cholecystitis and is generally associated with low complication and recurrence rates in suitable surgical candidates [3]. The clinical relevance of severity grading has been demonstrated, with outcomes correlating with disease severity [19]. Therefore, in high-risk patients, particularly elderly individuals with severe disease or significant comorbidities, operative risk is increased and alternative treatments such as gallbladder drainage are often selected [20]. PTGBD is known to be associated with a high recurrence rate of cholecystitis following tube removal, with previous studies reporting recurrence rates of approximately 41–46% [21,22]. Furthermore, although ET-GBD has been reported to have a lower recurrence rate of approximately 3%, long-term stent placement has been associated with an increased incidence of delayed adverse events, such as cholangitis [8].

The cholecystitis recurrence rate in the benign group was 2.5%, which is consistent with previous reports. In a study by Inoue et al. [8], the recurrence rate after EUS-GBD for calculous cholecystitis was reported to be 3.8%, which is comparable to the recurrence rate observed in our benign group. However, the study did not include or evaluate patients with cystic duct obstruction caused by cancer invasion or SEMSs.

The high recurrence rate in the malignant group may have been due to cystic duct obstruction. In patients with SEMSs, cystic duct obstruction was not caused by intraluminal stent occlusion but was primarily due to coverage of the cystic duct orifice or mechanical compression by the covered stent. In most malignant cases, cholecystitis arises from the invasion of the cystic duct by cancer or SEMS-related obstruction [23]. In such cases, cholecystitis may easily occur when the EUS-GBD route is obstructed by the cystic duct that has also been obstructed. Moreover, a weakened immune system due to cancer or chemotherapy can lead to infections, including recurrent cholecystitis [24]. Scheduled stent exchange may reduce the risk of recurrence and improve clinical outcomes in such patients. The median time to recurrence was 90 days in the malignant group. Therefore, in patients with a relatively favorable prognosis who are receiving chemotherapy, scheduled stent exchange approximately 3 months after EUS-GBD may be beneficial. Previous reports have shown a low recurrence rate of cholecystitis after EUS-GBD using lumen-apposing metal stents (LAMSs) [25,26], suggesting that the use of LAMSs may be advantageous for patients with malignant cystic duct obstruction. By contrast, in the benign group, even if the stent became dysfunctional, the cystic duct was expected to reopen naturally. In such cases, drainage with PS is sufficient and routine stent exchange is not necessary.

This study had some limitations. First, the potential for selection bias cannot be excluded because this was a retrospective study. In clinical practice, alternative drainage procedures such as ET-GBD or PTGBD may also be considered for high-risk patients. However, this study included only patients who underwent EUS-GBD, and therefore the selection of this procedure may have been influenced by physician preference, institutional expertise, or patient characteristics. Therefore, an evaluation of the superiority of EUS-GBD over other treatment methods could not be conducted. Furthermore, the retrospective design spanning more than a decade (2010–2023) likely introduced heterogeneity in patient selection, procedural techniques, and peri-procedural care. Device availability and management strategies have evolved during this period, but this was not accounted for in the analysis. Second, the shorter follow-up period in the malignant group likely reflects disease progression, which limits the assessment of long-term outcomes. Third, LAMSs could not be evaluated because it was not covered by insurance in Japan during the study period. Finally, owing to the small number of recurrent cases (*n* = 10), multivariate analysis was not feasible, and the findings should be interpreted with caution. Further prospective studies are warranted to validate these findings and to establish optimal stent management strategies, particularly in patients with malignancy-associated cholecystitis.

## 5. Conclusions

EUS-GBD is a safe and effective treatment for acute cholecystitis in patients who are not surgical candidates. However, patients with acute cholecystitis secondary to malignant cystic duct obstruction have a higher risk of recurrence. Careful follow-up and individualized management strategies, such as scheduled stent exchange or the use of LAMSs, are needed to optimize the outcomes in this high-risk group.

## Figures and Tables

**Table 1:** Baseline patient characteristics.

	All Patients (*n* = 139)	Malignant Group (*n* = 60)	Benign Group (*n* = 79)	*p* Value
Age (IQR), years	84 (74–89)	78 (67–85)	87 (78–91)	<0.0001
Sex, (male/female), *n*	72/67	32/28	40/39	0.752
ASA-PS classification ≥ 3, *n* (%)	71 (51)	19 (32)	52 (66)	<0.0001
Antithrombotic drugs, *n* (%)	44 (32)	10 (10)	34 (43)	0.0007
Severity of cholecystitis, *n* (%)				0.0069
Mild or moderate	130 (93)	60 (100)	70 (89)	
Severe	9 (7)	0 (0)	9 (11)	
Gallbladder stones, *n* (%)	60 (43)	11 (18)	49 (82)	<0.0001
Reasons for EUS-GBD, *n* (%)				
Cancer/SEMS/Calculous/Acalculous	32/28/77/2	32/28/0/0	0/0/77/2	
	(23/20/55/2)	(53/47/0/0)	(0/0/97/3)	
Follow-up duration (IQR), days	195 (57–392)	116 (29–224)	307 (93–522)	0.0002

*IQR*, interquartile range; *ASA-PS*, American Society of Anesthesiologists Physical Status; *EUS-GBD*, endoscopic ultrasound-guided gallbladder drainage; *SEMS*, self-expandable metallic stent.

**Table 2:** Procedural outcomes of EUS-GBD.

	All Patients (*n* = 139)	Malignant Group (*n* = 60)	Benign Group (*n* = 79)	*p* Value
Recurrence of cholecystitis, *n* (%)	10 (7.2)	8 (13.3)	2 (2.5)	0.015
Time to recurrence of cholecystitis (IQR), days	90 (18–361)	91 (17–302)	426 (19–833)	0.695
Early AEs, *n* (%)	15 (10.7)	8 (13.3)	7 (8.9)	0.214
Biliary peritonitis	11 (7.9)	7 (11.7)	4 (5.1)	0.153
Stent migration	4 (2.8)	1 (1.7)	3 (3.8)	0.457
Late symptomatic AEs except cholecystitis, *n* (%)	2 (1.4)	0 (0)	2 (2.5)	0.400
Cholangitis	2 (1.4)	0 (0)	2 (2.5)	0.400

*EUS-GBD*, endoscopic ultrasound-guided gallbladder drainage; *IQR*, interquartile range; *AE*, adverse event.

**Table 3:** Risk factors for recurrent cholecystitis after EUS-GBD.

	*n*	Number of Patients with Recurrent Cholecystitis	Univariate Analysis		
			OR (95% CI)		*p* Value
Age ≥ 84 years	62	2	0.28	(0.06–1.36)	0.115
Sex, male	72	4	0.60	(0.16–2.22)	0.442
Antithrombotic drugs	44	2	0.52	(0.11–2.55)	0.418
ASA-PS ≥ 3	71	2	2.08	(0.52–8.29)	0.166
Severe cholecystitis	9	0	N/A	N/A	N/A
malignant group	42	4	5.92	(1.21–29.01)	0.028
Gallbladder stones	67	4	0.7	(0.19–2.59)	0.589

*OR*, odds ratio; *CI*, confidence interval; *ASA*, American Society of Anesthesiologists; *N/A*, not applicable.

## Data Availability

The datasets used and/or analyzed in the current study are available from the corresponding author upon reasonable request.

## Supplementary Materials

The following supporting information can be downloaded at: https://www.mdpi.com/article/10.3390/jcm15103621/s1. Table S1. Details of recurrent cholecystitis.
