# Supplementary material for: Long-Term Outcomes of Endoscopic Ultrasound-Guided Gallbladder Drainage for Acute Cholecystitis in Non-Surgical Candidates: A Multicenter Retrospective Study
Source: J Clin Med. 2026 May 8;15(10):3621. doi: 10.3390/jcm15103621 (PMC13206763; doi:10.3390/jcm15103621)
Supplement: Supplementary file 1 [file jcm-15-03621-s001.zip › jcm-4238350-supplementary.pdf]

Table S1. Details of recurrent cholecystitis

No	Age	Sex	ASA-PS	Severity of initial cholecystitis	Reasons for EUS-GBD	Time to recurrence of cholecystitis, days
1	46	F	2	mild	Cancer invasion	350
2	71	F	2	mild	Cancer invasion	13
3	77	M	2	mild	Cancer invasion	157
4	75	F	3	moderate	Cancer invasion	31
5	75	M	2	moderate	Cancer invasion	10
6	85	M	2	moderate	Cancer invasion	150
7	71	F	1	moderate	SEMS	393
8	78	M	3	moderate	SEMS	30
9	67	F	2	mild	Calculous	19
10	89	F	3	moderate	Calculous	833

All patients improved after treatment for recurrent cholecystitis.
ASA-PS, American Society of Anesthesiologists Physical Status; *EUS-GBD*, endoscopic ultrasound-guided gallbladder drainage; *SEMS*, self-expandable metallic stent, *PTGBD,* percutaneous transhepatic gallbladder drainage.
